# Small Intestine Protection of Mica Against Non-Steroidal Anti-Inflammatory Drugs-Injury Through ERK1/2 Signal Pathway in Rats

**DOI:** 10.3389/fphar.2019.00871

**Published:** 2019-08-02

**Authors:** Shuo Zhang, Yinghua He, Zheng Shi, Jianping Jiang, Beihui He, Sumei Xu, Zhengyu Fang

**Affiliations:** ^1^Department of Gastroenterology, The First Affiliated Hospital, Zhejiang Chinese Medical University, Hangzhou, China; ^2^Department of Pharmacy, The First Affiliated Hospital of Zhejiang Chinese Medicine University, Hangzhou, China; ^3^Department of Preparation Center, The First Affiliated Hospital of Zhejiang Chinese Medicine University, Hangzhou, China; ^4^Laboratory of Digestive Disease, The First Affiliated Hospital of Zhejiang Chinese Medical University, Hangzhou, China; ^5^Department of General Family Medicine, The First Affiliated Hospital of Zhejiang Chinese Medicine University, Hangzhou, China; ^6^Department of Proctology, The First Affiliated Hospital of Zhejiang Chinese Medicine University, Hangzhou, China

**Keywords:** mica, diclofenac, small intestinal injury, PAR-2/ERK pathway, non-steroidal anti-inflammatory drugs

## Abstract

**Objective:** The impact of non-steroidal anti-inflammatory drugs (NSAIDs) to damage the small intestine has been well known. Mica, one kind of natural clay, has been widely marketed in China for the treatment of gastric diseases. However, the role and mechanism of mica in small intestinal injure is still unknown. The study was designed to declare the effects of mica on intestinal injury induced by diclofenac in rats.

**Methods:** Rats were randomly divided into control, model, PAR-2 agonist group (SLIGRL-NH2group), control peptide group (LRGILS-NH2 group), and ERK blocker group (eight mice per group). Morphological changes of mucous membrane of small intestine were observed, and the expression of tryptase, PAR-2, and p-ERK1/2 was measured by immunohistochemistry and western blot. PAR-2 mRNA was tested by qRT-PCR. Rats were also randomly divided into control, model, and mica group (eight mice per group). Morphological changes of mucous membrane were observed. The expression of tryptase, PAR-2, and p-ERK1/2 was measured by immunohistochemistry.

**Results:** The expression of trypsin, PAR-2, and p-ERK1/2 was increased in model group compared with control. The expression of PAR-2 and p-ERK1/2 was increased in SLIGRL-NH2 group compared with model, but not LRGILS-NH2 group. The expression of PAR-2 was down-regulated in ERK blocker group compared with SLIGRL-NH2 group. Macroscopically visible lesions of mucous membrane were positively correlated with the expression of PAR-2 and p-ERK1/2. Furthermore, we also found that mica could inhibit small intestinal injure, as evidenced by the improvement of macroscopic damage. Tryptase, PAR-2, and p-ERK1/2 expression was down-regulated in mica group compared with model group.

**Conclusion:** Mica inhibit small intestinal injury induced by NSAIDs *via* ERK signaling pathway.

## Introduction

Non-steroidal anti-inflammatory drugs (NSAIDs) such as diclofenac and aspirin are commonly used in the clinic for antipyretic and analgesic. With the increasing application of NSAIDs and the popularization of capsule endoscopy and double balloon enteroscopy, the side effects on digestive tract including small intestinal injury have received increasing attention (Silverstein et al., 2000; Schnitzer et al., 2004; Kameda et al., 2008; Sugimori et al., 2008; Watanabe et al., 2008). Maiden et al. (2005) have confirmed that taking diclofenac sodium for only 2 weeks would cause small intestinal injury in 68–75% healthy people. Small intestinal injury induced by NSAIDs is characterized by intestinal mucosal erosion, ulceration, and even bleeding (Fornai et al., 2016). However, the task of prevention and treatment of the digestive tract side effects caused by NSAIDs is very arduous in China because of large population. Small intestinal injury is still one of the urgent issues to be solved in the safe application of NSAIDs in the areas of chronic pain, tumor chemoprevention, and cardio-cerebral vascular disease. Therefore, clarifying the pathogenesis of NSAIDs enteropathy has great clinical significance in the prevention and treatment of NSAID-inducing intestinal injury, resulting in great social and economic benefits.

Protease activated receptor (PAR)-2, which belongs to the PAR family, is a seven-transmembrane G protein coupled receptor (GPCR) and is widely distributed in the body. Intestinal administration of PAR-2 activator causes rapid development of intestinal inflammation, change of capsaicin-sensitive neurons, nitric oxide, and cell permeability (Cenac et al., 2003). In intestinal epithelial cells, PAR-2 forms a complex with the connexin β-arrestin (β-ARR), which mediates endothelial cell receptors and regulates them *via* the ERK1/2 signaling pathway (Mccoy et al., 2010). The ERK1/2 pathway is demonstrated to be involved in the regulation of the cytoskeleton, which affects intestinal permeability (Hyun et al., 2010). Thus, it can be seen that in the pathogenesis of NSAIDs, ERK1/2 pathway may play a crucial part in the changes of intestinal pathophysiological, including changes in intestinal permeability and intestinal motility patterns, release of inflammatory mediators, and ion transport disorders.

Mica has been applied to various acute or chronic inflammations of the digestive tract in our hospital for nearly 30 years due to its good clinical efficacy and safety. Previous studies have verified that mica could reduce the intestinal permeability of rat and has a protective effect on the damage of intestinal mucosal caused by NSAIDs (Chao and Zhang, 2012). However, the relationship between mica and PAR-2 or ERK1/2 pathway remains unknown. To explain these important issues, we projected the study to illuminate whether mica modulated PAR-2 expression and activated ERK1/2 signaling pathway to improve the symptoms of small intestinal disease that diclofenac induced.

## Methods

### Animals

Eight-week-old male Sprague-Dawley rats (200 ± 20 g), which were purchased from Zhejiang Chinese Medicine University, were fed normal laboratory chow and tap water. Rats were randomly divided into several groups. They were housed, four subjects in a cage, in temperature-controlled rooms on a light/dark (12 h/12 h) cycle at 23°C and 50–60% humidity. The study obtained the approval of the local Animal Ethic Committee of Zhejiang Chinese Medicine University.

### Diclofenac-Induced Small Intestinal Injury

Intestinal injury was induced by diclofenac on the basis of the method previously developed in our laboratory (Chao and Zhang, 2012). The treatment course and dose of diclofenac were adopted in order to acquire small intestinal injuries induced by NSAIDs in humans. To achieve this aim, non-fasted rats (n = 8 rats for each group) were treated for 5 days with diclofenac 7.5 mg/kg, 2/day, by a single intragastric administration. The control groups received the same dose of saline.

### Treatment

The mica (120 mg/kg/day), which was supplied from Zhejiang Chinese Medicine University, was administered 9 days before diclofenac-induced, with concomitant treatment with diclofenac on the final 5 days.

### Inhibitor and Activator Treatment

After establishing the NSAID models, the SLIGRL-NH2 (or LRGILS-NH2) group was injected a single dose (3 μmol/kg) of SLIGRL-NH2 (or LRGILS-NH2) (Beyotime, China) through tail vein injection. The eight SD rats in ERK blockers group were treated a single dose of SL327 (Beyotime, China) 10 min prior to SLIGRL-NH2 injection, with 50 mg/kg. Twenty-four hours after drug treatment, rats were killed under ether anesthesia, and the small intestines were taken for observation and kept with 2% formalin for fixation of the tissue.

### Immunohistochemistry Staining and Evaluation of PAR-2 and P-ERK1/2 Expression

Tissues of small intestine (2–3 cm) were fixed with 70% ethanol overnight and then embedded in paraffin. The tissues were cut into sections at the thickness of 4–6 μm. Process of immunohistochemistry staining was operated as previously described (Ordonez, 2003). After series of treatments, tissue slices were immediately incubated with normal serum for 30 min, rabbit polyclonal antibody against PAR-2, p-ERK1/2, tryptase (1:500, Abcam, UK) at 4°C overnight, and then incubated with horseradish peroxidase anti-rabbit secondary antibody (Boster, 72331B) for 30 min. The expression of PAR-2, p-ERK1/2, and tryptase with development of brown color was confirmed by diaminobenzidine tetrahydrochloride solution (DAB, Cowin Bioscience, China) staining. All sections were taken three to five photos under a fluorescence microscope to recode the positive area and positive index.

### Western Blot Assay

Intestine tissues of rats were lysed on ice by protein extraction solution (Beyotime, China). Protein concentration of solution was detected by a BCA protein assay kit (Boster, China). Total proteins (20–50 μg) per sample were operated by SDS-PAGE and blotted to the membrane of PVDF (Millipore, USA).The PVDF membrane blocking with 5% milk was incubated for 6 h with primary antibodies that diluted in Dilution Buffer (Beyotime, China) with proper concentration. Rabbit monoclonal antibody recognizing PAR-2, p-ERK1/2, and ERK1/2 (1:500, Abcam, UK) were diluted at 1:500. After washing in TBS/Tween-20 solution (Boster, China), blot membranes were incubated in conjugate antibody of goat anti-rabbit horseradish peroxidase (Bioworld, USA) in TBS/Tween-20 solution with 5% skim milk for 1 h. Protein blots were measured by protein blots detection system (ChemidocXRS, Bio-RAD, USA) after washing three times with TBS/Tween-20 solution. The variation of stripe concentration was revealed as fold changes compared to the normalized β-actin or the total proteins. Representative bands were selected from the three independent experiments.

### Real-Time Quantitative RT-PCR

Total RNA was extracted from intestine tissues of rats using TRIzol (Invitrogen, USA) according to the reagent instruction. Primer sequences were designed using Primer Premier 5.0. For RT-PCR, the following primer sequences were used: PAR-2 (forward: 5’-AACATCACCACCTGTCACGA-3’; reverse: 5’-CACGTAGGCAGACGCAGTAA’). β-Actin was used as an internal reference for comparison with the target product, and RT-PCR was used to detect the changes in the expression of PAR-2 mRNA in intestinal epithelial cells. The results were repeated at least three times independently.

### Statistical Analysis

All values were analyzed by the Soft of SPSS 17.0 and expressed as mean ± SEM. The statistical significance of data sets was measured with Student unpaired *t* test for comparison between means or one-way analysis of variance (ANOVA). ^#^ *p* or *p < 0.05 and **p < 0.01 were considered to be statistically significant.

## Results

### Relationship Between the PAR-2 and P-ERK1/2 Expression With Nsaid-Induced Small Intestinal Injury

#### Expression of PAR-2 and p-ERK1/2 in nsaid-Induced Small Intestinal Injury.

**Figure 1:**
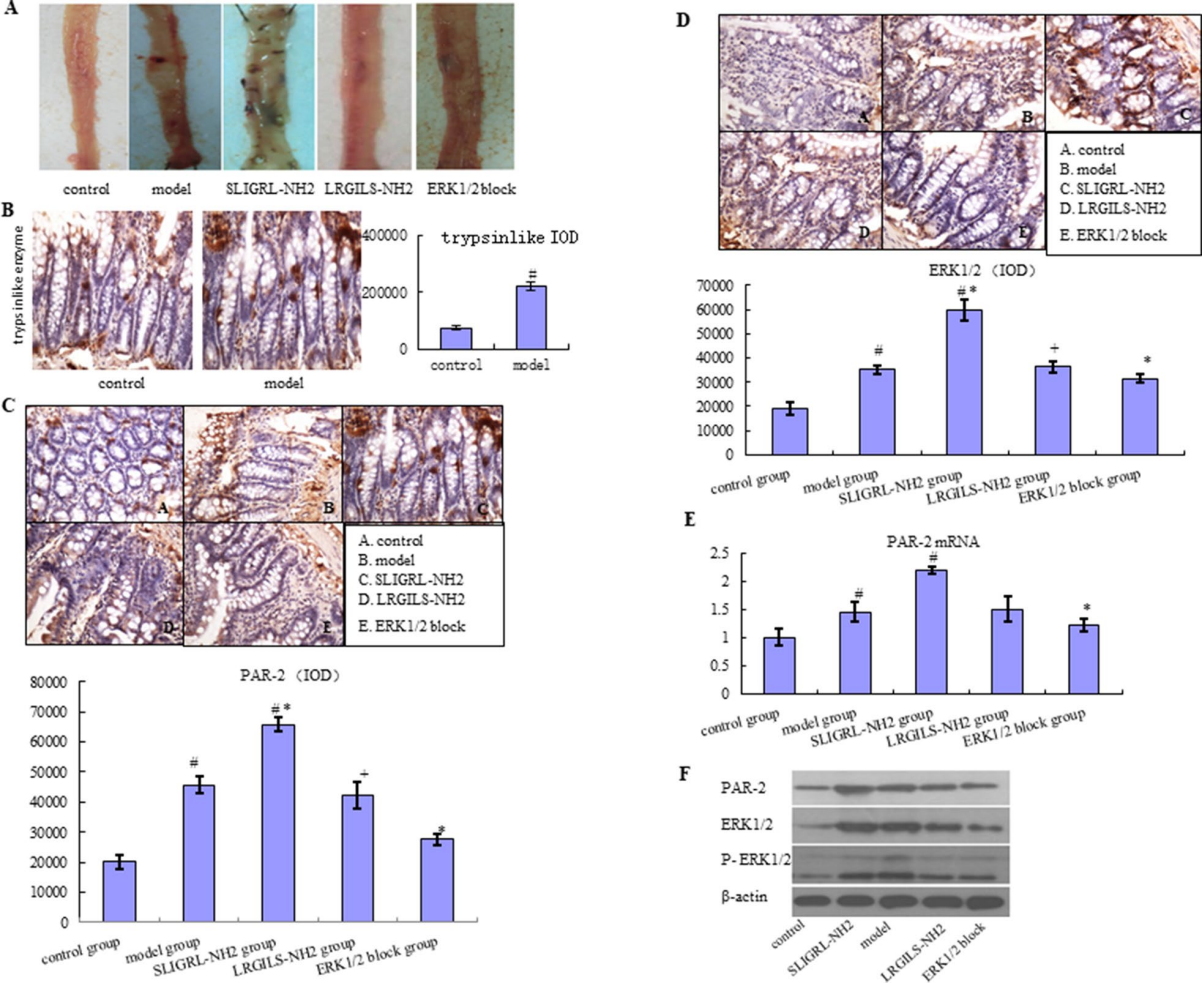
Relationship between the PAR-2 and p-ERK1/2 expression with NSAID-induced small intestinal injury. **(A)** Macroscopic observation of small intestinal injury in control group, model group (diclofenac, 7.5 mg/kg BID, 5 days), SLIGRL-NH2 group (PAR-2 agonist group), LRGILS-NH2 group (PAR-2 antagonist group), and ERK blocker group. **(B)** The protein expression of tryptase was measured in control and model group by IHC (×400). The protein expression of PAR-2 **(C)** and p-ERK1/2 **(D)** was measured in control, model, SLIGRL-NH2, LRGILS-NH2, and ERK blocker group by IHC (×400). **(E)** The mRNA levels of PAR-2 were detected in control, model, SLIGRL-NH2, LRGILS-NH2, and ERK blocker group by qRT-PCR. **(F)** The protein expression of PAR-2 and p-ERK1/2 in control, model, SLIGRL-NH2, LRGILS-NH2, and ERK blocker group was measured by western blot. The bar graphs (mean ± SD) and representative images are shown. ^#^ *p* < 0.05 vs the control group, ^*^ *p* < 0.05 vs the model group. Representative views from each group are presented.

In the small intestine from control group, macroscopic examination did not form any lesion. But administration of diclofenac and SLIGRL-NH2 was associated with the occurrence of visible hyperemia, erosion, and ulcer in mucosa (**Figure 1A**). What is more, damage was more severe in SLIGRL-NH2 group, but not LRGILS-NH2 group compared to model. To declare the different expression about the PAR-2 and p-ERK1/2 in control, model, SLIGRL-NH2, and LRGILS-NH2 group, the expression level of PAR-2 and p-ERK1/2 was determined by IHC (**Figure 1C, D**) and western blot (**Figure 1F**). The mRNA expression level of PAR-2 was determined by qRT-PCR (**Figure 1E**). The result showed that the protein level of PAR-2 and p-ERK1/2 was increased in tissues of model group compared with the control. Likewise, PAR-2 was increased in SLIGRL-NH2 group, but not LRGILS-NH2 group compared to model. It indicated that PAR-2 activation aggravated the damage of intestine. Meanwhile, tryptase was also up-regulated in model group analyzed by IHC (**Figure 1B**). There results suggested that the expression of PAR-2 and p-ERK1/2 was positively correlated with NSAID-induced small intestinal injury.

#### NSAID-Induced Small Intestinal Injury is Prevented by Blocking p-ERK1/2

The expression level of PAR-2 and p-ERK1/2 was increased in SLIGRL-NH2 group with enhanced small intestinal injury compared with model group, but decreased in ERK blocker group with improvement of small intestinal injury according to IHC, qRT-PCR, western blot, and macroscopic examination (**Figure 1**). These results performed that NSAID-induced small intestinal injury can be prevented by blocking the expression of p-ERK1/2. PAR-2 could active the phosphorylation of ERK1/2 in NSAID-induced small intestinal injury.

### Effects of Mica on NSAID-Induced Small Intestinal Injury

#### Macroscopic Exhibition of the Intestine

**Figure 2:**
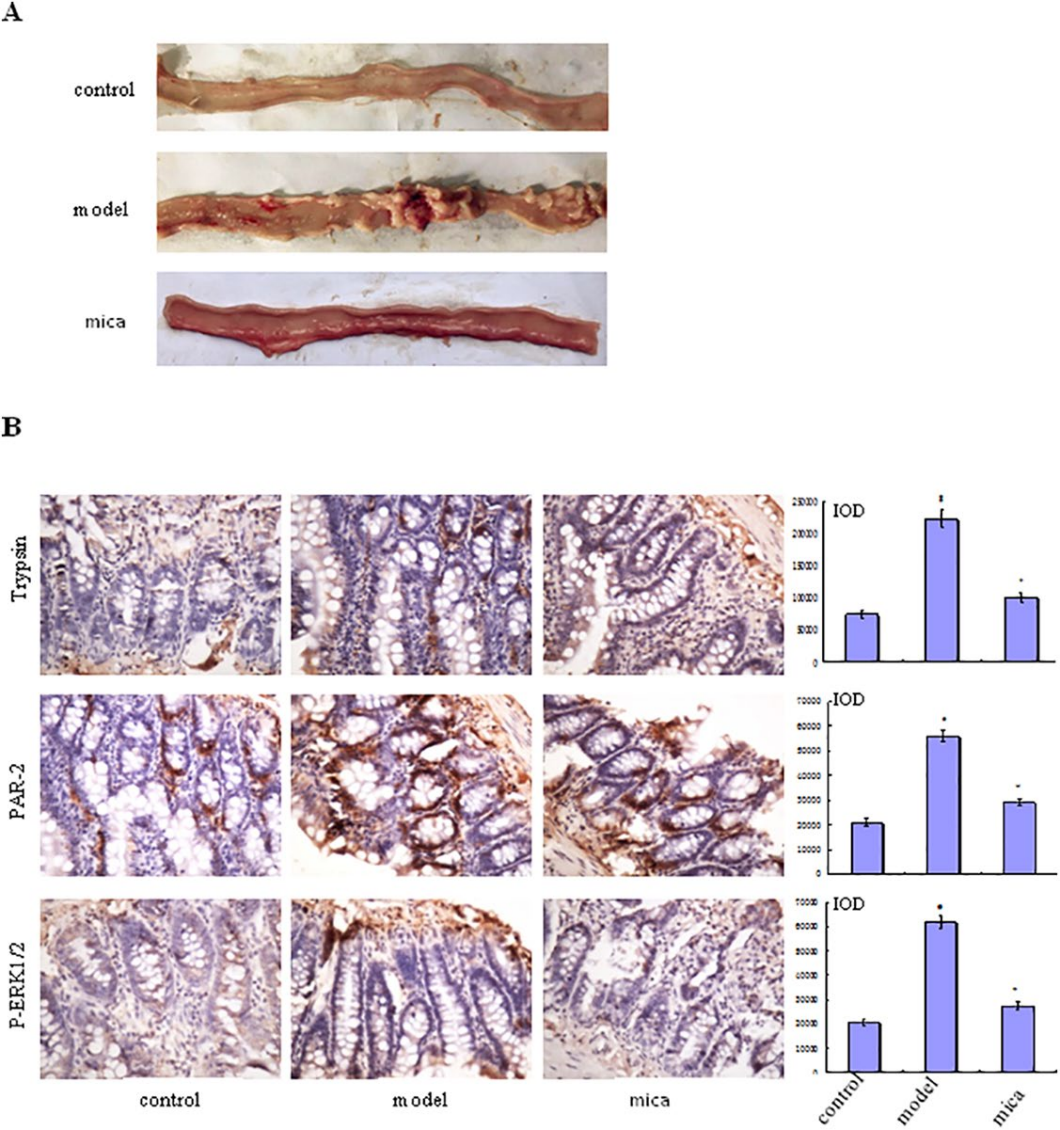
Effects of mica on NSAID-induced small intestinal injury. **(A)** Macroscopic observation of small intestinal injury in control, model, and mica group (120 mg/kg/d, 9 days). **(B)** The protein expression of tryptase, PAR-2, and p-ERK1/2 was measured in control, model, and mica group by IHC (×400). The bar graphs (mean ± SD) and representative images are shown. ^#^ *p* < 0.05 vs the control group,^*^ *p* < 0.05 vs the model group. Representative views from each group are presented.

To illuminate the effects of mica on NSAID-induced small intestinal injury, rats with diclofenac-induced small intestinal injury was treated with mica. We found that small intestinal mucosa of mica group has slightly edema, scattered erosion, and a few shallow ulcers compared with model group in which erythema, erosion, and more ulcers were covered (**Figure 2A**).

#### Mica Inhibits the Progress of NSAID-Induced Small Intestinal Injury

Immunohistochemical examination of small intestine tissue from model group showed higher levels of tryptase, PAR-2, and p-ERK1/2 expression than those of control group (**Figure 2B**). In mica group, the levels of tryptase, PAR-2, and p-ERK1/2 were decreased compared with model group. This result suggested that mica inhibited the progress of NSAID-induced small intestinal disease through or partly by ERK signal pathway, PAR-2 being decreased simultaneously.

## Discussion

The intestine mucosa is the important barrier consisting of intestinal epithelial cells and the connection with adjacent intestinal epithelial cells. Integrated structure and function are significant in maintaining the body’s homeostasis. As is well known, NSAIDs induce injury in gastroduodenal. NSAID-induced small intestinal mucosal injury should also be taken seriously. The pathogenesis of intestinal damage is still not completely understood. Mechanisms of NSAID-related damage include the activities of prostaglandin-endoperoxide synthase 1 (PTGS1 or COX1) and PTGS1 (COX2) (Bjarnason et al., 2018). In addition, the absence of acid and the presence in the intestinal lumen of bacteria and bile may trigger specific NSAID-related pathogenic mechanisms (Scarpignato, 2008). Study found that intestinal epithelial permeability increased and a large number of mast cells assembled during inflammation occurring in the small intestine (Zhao et al., 2018). In animal models without mast cells or with mast cell stabilizers, chronic stress, allergic inflammation, parasitic infection, and chemotherapy-induced intestinal injury were significantly reduced (Soderholm et al., 2002). Tryptase, which is the secretion of activated mast cells, activates PAR-2 (Coelho et al., 2002).

PAR-2 is one of the four different PARs that is currently known and belongs to the family of GPCRs. Past research has suggested that PAR-2 has extensive biological effect in intestinal inflammation and damage. Liu et al. proved that intestinal ischemia–reperfusion injury was suppressed by cromolyn sodium *via* inhibiting PAR-2 expression in rats (Liu et al., 2012). In terms of intestinal inflammation, the role of PAR-2 receptor is proinflammatory and appears as a potential therapeutic target for inflammatory intestinal disease treatments (Patel et al., 2010). The ERK1/2 pathway is related to the regulation of cytoskeleton, which affects the permeability of intestinal wall (Hyun et al., 2010). Our study showed that the expression of tryptase and PAR-2 increased significantly in the small intestinal mucosa when the NSAID-inducing intestinal injury occurred. Further use of PAR-2 activator (SLIGRL-NH2) showed that ulcers and even perforation increased in small intestinal mucosal injury compared with model group. This study also found that blocking ERK1/2 could greatly reduce intestinal damage in rats with NSAIDs induced and caused by PAR-2 activator if ERK blocker was administered in advance, suggesting that PAR-2 may damage intestinal mucosa by activating ERK1/2 pathway. But the specific role of PAR-2 in intestinal damage needs to do much deeper research in the future, such as study using PAR-2 inhibitor.

There is currently no method for effectively preventing and treating NSAID small intestinal injury. For example, misoprostol can reduce intestinal permeability, but it needs large doses to prevent NSAID intestinal diseases. The effect is not satisfactory because of valuableness and adverse reactions. The combination of sulfasalazine and NSAIDs can significantly reduce intestinal inflammation and chronic blood loss caused by the latter, but long-term use should consider the inhibition of liver and bone marrow. Mica has been used in various clinical departments of our hospital for nearly 30 years of acute or chronic gastrointestinal tract inflammation because of its good clinical efficacy and safety. Studies (Tognetto et al., 2000; Kawabata et al., 2001; Gaca et al., 2002; Nishikawa et al., 2002) had demonstrated that mica could inhibit gastric ulcer, chronic atrophic gastritis, and ulcer active colitis of experimental rat. But the exact mechanism about small intestine protection of mica is unclear. Interestingly, in the present study, we found that mica could effectively treat NSAID-inducing small intestinal injury, and its mechanism may reduce the activation of PAR-2 and inhibit the intestinal mucosal tryptase *via* ERK1/2 pathway. On the other hand, this study provides a further experimental basis for the treatment of NSAID-inducing small intestinal injury with traditional Chinese medicine. It is necessary to further study the long-term efficacy of mica on intestinal damage caused by NSAIDs.

## Data Availability

The raw data supporting the conclusions of this manuscript will be made available by the authors, without undue reservation, to any qualified researcher.

## Ethics Statement

This study was carried out in accordance with the recommendations of the local Animal Ethic Committee of Zhejiang Chinese Medicine University. The protocol was approved by the local Animal Ethic Committee of Zhejiang Chinese Medicine University.

## Author Contributions

SZ and YH designed the experiment. ZS, JJ, BH, and YH performed the experiment. SX and ZF analyzed the date. YH wrote the paper. SZ revised the paper.

## Funding

A portion of this work was supported by the National Natural Science Foundation (81573760) and Zhejiang Province undergraduate academic leaders of young academic climbing project (pd2013209). Funding was provided by Zhejiang Provincial Natural Science Foundation of China under Grant No. LY18H030001, The Medicine and Health Science and Technology Plan Projects in Zhejiang province (2017KY413), Traditional Chinese Medicine Science and Technology Plan of Zhejiang Province (2017ZA089, 2016ZB071, 2015ZZ012, 2014ZA030), Medical Health Platform Plan Projects of Zhejiang Province (2015RCA020), and Zhejiang Provincial Natural Science Foundation of China (LY16H030010).

## Conflicts of Interest Statement

The authors declare that the research was conducted in the absence of any commercial or financial relationships that could be construed as a potential conflict of interest.
